# Navigating Cirrhosis: A Comprehensive Review of Liver Scoring Systems for Diagnosis and Prognosis

**DOI:** 10.7759/cureus.57162

**Published:** 2024-03-29

**Authors:** Palash S Kotak, Jayanth Kumar, Sunil Kumar, Anuj Varma, Sourya Acharya

**Affiliations:** 1 Internal Medicine, Jawaharlal Nehru Medical College, Datta Meghe Institute of Higher Education & Research, Wardha, IND

**Keywords:** precision medicine, interdisciplinary collaboration, prognosis, diagnosis, liver scoring systems, cirrhosis

## Abstract

This comprehensive review navigates the landscape of liver scoring systems for the diagnosis and prognosis of cirrhosis. Cirrhosis, a chronic and progressive liver disease, presents significant challenges in its diagnosis and management. The review begins by defining and providing an overview of cirrhosis, emphasizing its clinical implications. Highlighting the significance of liver scoring systems, including the Child-Pugh score, end-stage liver disease, albumin-bilirubin (ALBI) score, and fibrosis-4 (FIB-4) index, the study explores their role in assessing liver dysfunction severity and predicting outcomes. A meticulous analysis identifies the strengths and limitations of these scoring systems, offering valuable insights for clinicians. The recommendations emphasize incorporating these tools into routine clinical practice for early intervention and personalized treatment plans. Interdisciplinary collaboration is underscored as crucial for a holistic approach to cirrhosis management. The conclusion calls for future research to refine existing scoring systems, explore emerging biomarkers and imaging techniques, and conduct prospective studies to enhance precision. By embracing these recommendations, the medical community can advance the understanding and management of cirrhosis, ultimately improving patient outcomes and revolutionizing liver disease approaches.

## Introduction and background

Cirrhosis represents the advanced stage of liver fibrosis, where fibrous scar tissue gradually replaces the normal liver tissue [1]. This structural distortion compromises the liver's ability to function correctly, leading to impaired blood flow, disruption of metabolic processes, and potential complications such as portal hypertension, ascites, and hepatic encephalopathy. As a condition with significant morbidity and mortality, cirrhosis poses a considerable burden on global healthcare systems [2].

The severity and prognosis of cirrhosis can vary widely among individuals, necessitating accurate tools for assessment and stratification. Liver scoring systems play a pivotal role, offering a quantitative means to evaluate the degree of liver dysfunction and predict the likelihood of complications or mortality [3]. These scoring systems are invaluable clinical tools for healthcare professionals to make informed decisions regarding patient management, treatment strategies, and transplant eligibility [4].

This review aims to critically and comprehensively analyze existing liver scoring systems utilized in the diagnosis and prognosis of cirrhosis. By exploring the intricacies of these scoring systems, we aim to provide healthcare practitioners, researchers, and clinicians with a thorough understanding of their strengths, limitations, and comparative effectiveness. Furthermore, this review highlights the evolving landscape of liver disease assessment, discussing emerging trends and future directions.

## Review

Importance of early diagnosis

Challenges in Detecting Cirrhosis

Early detection of cirrhosis is imperative due to the condition's tendency to remain asymptomatic until decompensation occurs, significantly increasing the risk of mortality. Physicians must inquire about risk factors predisposing patients to cirrhosis, and if there is a high clinical suspicion for liver disease, further serologic work-up should be initiated promptly. Abdominal ultrasonography is a specific, reliable, non-invasive, rapid, and cost-effective test that should be the initial radiographic study for diagnosing cirrhosis [5]. Experiences and perceptions among general practitioners (GPs) suggest that early intervention can effectively address all significant causes of liver disease. Nevertheless, identifying the disease in high-risk groups presents challenges, highlighting the need for support to enhance awareness, knowledge, and confidence among GPs regarding the early detection of liver disease [6]. Timely diagnosis of cirrhosis, particularly during the compensated stage, holds paramount importance in improving patient outcomes and mitigating mortality risks [7].

Role of Liver Scoring Systems in Early Diagnosis

The significance of liver scoring systems in facilitating early diagnosis and predicting mortality in decompensated liver cirrhosis cannot be overstated. Various scoring systems, including the Child-Turcotte-Pugh (CTP) score, model for end-stage liver disease (MELD) score, MELD-Na, and MELD to serum sodium ratio (MESO), have been instrumental in assessing the severity of liver disease and forecasting prognosis [8,9]. By leveraging these scoring systems, clinicians can make more accurate diagnoses, tailor treatment strategies effectively, and ultimately improve patient outcomes. However, the current models may benefit from incorporating more sensitive indicators or developing newer, more precise models to optimize mortality prediction in decompensated liver cirrhosis [8]. Furthermore, it is crucial to recognize that liver scoring systems should complement rather than replace clinical judgment. Therefore, further research efforts, including multicenter studies with larger sample sizes and long-term follow-ups, are warranted to enhance the accuracy and reliability of these scoring systems [8,9]. While liver scoring systems play a critical role in predicting mortality in liver cirrhosis, continual refinement and improvement are necessary to maximize their effectiveness in early diagnosis and prognosis assessment.

Impact on Treatment Outcomes

**Figure 1:**
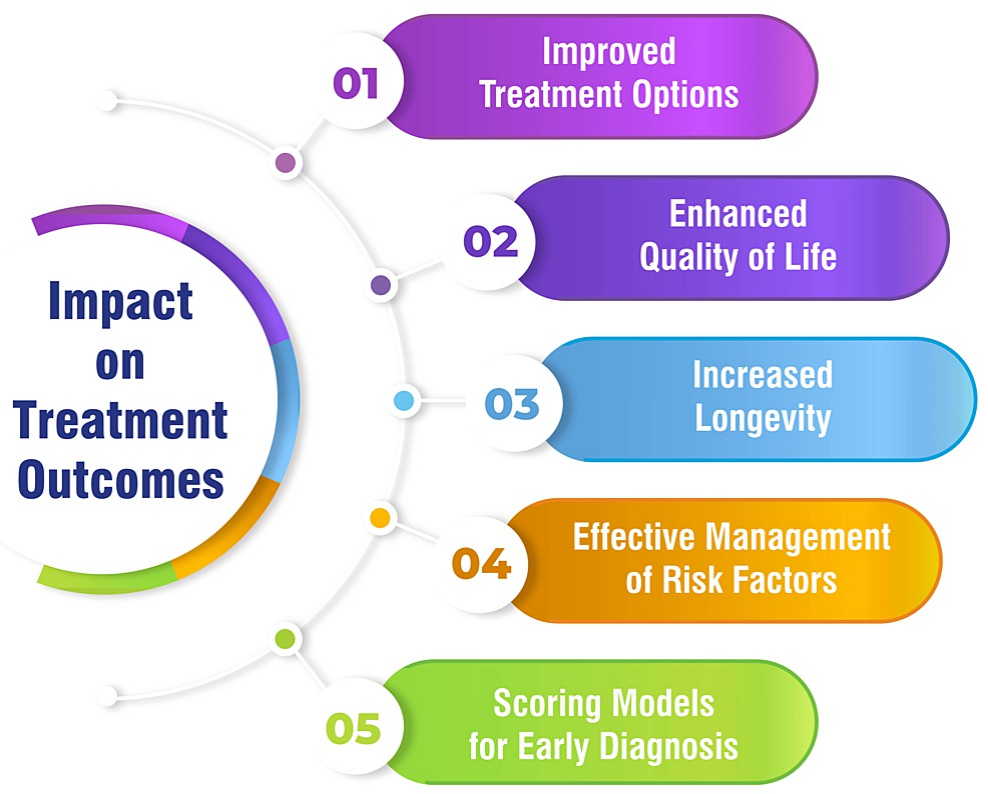
Impact on treatment outcomes This figure is self-created by the corresponding author.

Early detection of liver cancer offers many benefits, starting with improved treatment options and outcomes. When liver cancer is diagnosed in its early stages, before spreading to other organs, it becomes more amenable to successful treatment. Early-stage liver cancer can often be effectively addressed through curative interventions such as surgery, liver transplantation, or localized therapies like radiofrequency ablation [10]. This early intervention not only increases the likelihood of successful treatment but also reduces the need for more aggressive approaches, thereby enhancing the quality of life for patients. Early-stage liver cancer treatment typically entails milder side effects and shorter recovery periods, contributing to an overall improved quality of life [10]. Moreover, timely detection of liver cancer extends beyond immediate treatment benefits; it also holds the potential to increase longevity significantly. By diagnosing liver cancer early and initiating appropriate treatment promptly, individuals have a better chance of achieving long-term survival and effectively managing the disease over time [10]. This underscores the critical role of early detection in mitigating the impact of liver cancer and maximizing patient outcomes. Furthermore, early detection facilitates the effective management of underlying risk factors, particularly in individuals with cirrhosis who are at heightened risk of developing liver cancer. Regular screenings for early detection enable proactive management of the disease, allowing healthcare providers to implement interventions that can help mitigate disease progression. For instance, individuals with non-alcoholic fatty liver disease (NAFLD) are at risk of liver inflammation and, in some cases, progression to liver cancer. Early detection through screening empowers healthcare providers to intervene early, effectively managing the disease and minimizing its potential consequences [10]. Additionally, advancements in medical research have led to the development of scoring models specifically tailored for the early diagnosis of infection in liver failure patients. These studies have demonstrated high overall accuracy and good reproducibility, highlighting the potential of scoring models as valuable tools in the early detection and management of liver diseases [11]. This underscores the importance of ongoing research efforts in developing innovative approaches to enhance early diagnosis and improve patient outcomes in individuals at risk of liver cancer and related conditions. Impact on treatment outcomes are shown in Figure 1.

Liver scoring systems

Child-Pugh Score

The Child-Pugh score is a scoring system for assessing the severity of liver disease and predicting mortality among patients with cirrhosis [12-14]. Comprised of five clinical measures, including serum bilirubin, serum albumin, prothrombin time, ascites, and hepatic encephalopathy, this score is calculated based on a scale ranging from one to three for each parameter, with three indicating the most severe condition [12-14]. The cumulative score, ranging from five to 15, categorizes the severity of cirrhosis into three classifications: Child-Pugh A (five to six points), Child-Pugh B (seven to nine points), and Child-Pugh C (10-15 points) [12-14]. Utilizing an online calculator or manually adding scores for each parameter facilitates the calculation of the Child-Pugh score [12-14]. Although the Child-Pugh score provides insight into the severity of liver disease, its limitations include subjective assessment requirements for grading ascites and encephalopathy and the lack of consideration for renal function [12-14]. Consequently, it is recommended to view the Child-Pugh score as a supplementary tool rather than a replacement for clinical judgment [14-16].

Model for End-Stage Liver Disease

Components and calculation: The MELD score is a crucial tool for assessing the severity of liver disease and predicting mortality in patients with cirrhosis [17]. Computed using serum bilirubin, serum creatinine, and international normalized ratio (INR), the MELD score ranges from six to 40, with higher scores indicating more severe liver disease [9,17]. Calculation of the MELD score involves a specific formula: MELD(i)=round1 (0.378*loge(bilirubin))+(1.120loge(INR))+(0.957loge(creatinine))+0.643 1 rounded to the tenth decimal place. MELD=MELD(i)+1.32*(137-Na)-(0.033MELD(i)(137-Na)). The upper limit of serum sodium (Na) is capped at 137, and the upper limit of serum creatinine is capped at four. In cases where the patient underwent dialysis at least twice in the past week, serum creatinine is automatically adjusted to 4.0. The maximum MELD score attainable is 40 [17]. While the MELD score is effective in predicting mortality in liver cirrhosis patients, it may have prognostic errors in predicting death due to extrahepatic organ dysfunction [9]. Additionally, other general scoring systems such as Acute Physiology and Chronic Health Evaluation (APACHE) II and III, Sequential Organ Failure Assessment (SOFA), Multiple Organ Dysfunction Score (MODS), and RIFLE (Risk, Injury, Failure, Loss And End-Stage Renal Failure) can be utilized in hospitalized cirrhosis patients [9].

Use in transplantation: The MELD score plays a pivotal role in prioritizing liver transplant candidates aged 12 and above based on the severity of their liver disease [18]. It estimates the likelihood of a patient surviving their disease over the next three months [19]. Similarly computed using serum bilirubin, serum creatinine, and INR, the MELD score ranges from six to 40, with higher scores indicating more severe liver disease [18,20]. Patients with higher MELD scores are given higher priority on the transplant waitlist for a deceased donor organ [21]. Validated as a measure of the probability of mortality within three months in transplant patients with end-stage chronic liver disease, the MELD score has been instrumental in allocating livers for transplantation since 2002 [22]. This implementation has led to a reduction of 3.5% in waiting list mortality, a 10.2% increase in deceased donor transplants, and a 12% decrease in patients awaiting transplantation [22]. Notably, patients with lower MELD scores should still be considered for liver transplantation, as significant living-donor liver transplants can serve as life-saving options for this subset of patients who may otherwise not qualify for transplantation [20].

Albumin-Bilirubin Score

Components and calculation: The albumin-bilirubin (ALBI) score represents a straightforward and objective scoring system utilized to evaluate liver function and prognosticate outcomes for patients with liver disease, particularly hepatocellular carcinoma (HCC) [23-25]. Calculation of the ALBI score involves the formula: ALBI score=(log10 bilirubin [µmol/L]×0.66)+(albumin [g/L]×−0.0852) [23,24]. Ranging from -2.60 to 2.59, lower ALBI scores correspond to better liver function [25]. Offering an alternative to the traditional Child-Pugh grade, the ALBI score has demonstrated comparable performance without necessitating subjective variables [25,26]. Easily obtained through a non-invasive blood test, the ALBI score is objectively assessed [24]. Its validity has been confirmed across studies involving HCC patients, effectively stratifying individuals into distinct prognostic groups [24,26,27].

Comparative analysis with Child-Pugh and MELD: The Child-Pugh and MELD scores are instrumental in evaluating the prognosis of liver cirrhosis. A systematic review and meta-analysis revealed that the MELD score exhibited a smaller negative likelihood ratio and higher sensitivity than the Child-Pugh score. However, the latter demonstrated higher specificity within specific patient subgroups [28]. Another study examined the predictive capabilities of the Child-Pugh, MELD, and FIB-4 scores in hepatitis C virus-infected individuals, highlighting the strong predictive ability of the MELD score in severe posthepatectomy liver failure [29]. Furthermore, a comprehensive review concluded that while the prognostic values of the Child-Pugh and MELD scores were generally similar, their utility might vary across specific clinical conditions, necessitating further investigation into their respective indications [28]. Thus, both scoring systems possess distinct strengths and limitations, emphasizing the importance of tailoring their use to the individual patient's clinical context.

Fibrosis-4 Index

Components and calculation: The Fibrosis-4 (FIB-4) index is a non-invasive scoring system designed to estimate the degree of liver fibrosis. Comprising four parameters, age, aspartate aminotransferase (AST), platelet count, and alanine aminotransferase (ALT), the FIB-4 index is calculated using the formula: FIB-4 index=(Age (years)xAST (U/l))/(Platelet count (10^9/l) x ALT^0.5 (U/l)). Ranging from 0.22 to 7.7, higher FIB-4 index values correlate with more advanced fibrosis stages. Demonstrating utility beyond fibrosis estimation, the FIB-4 index has proven effective in predicting HCC development among patients with concurrent NAFLD and chronic hepatitis B. Additionally, it has been utilized to forecast mortality in individuals with liver cirrhosis. Because of its simplicity and non-invasive nature position, the FIB-4 index is a valuable screening tool for patients suspected to be at risk for liver fibrosis [30-32].

Application in non-invasive assessment of fibrosis: The FIB-4 index is crucial in assessing liver fibrosis across various clinical contexts, including chronic hepatitis C (CHC) and hepatitis B (CHB). Demonstrating favorable diagnostic accuracy and high positive predictive value in CHC patients, the FIB-4 index has been instrumental in identifying fibrosis severity. In CHB, a cut-off ≤1.45 has been utilized to distinguish between moderate and severe fibrosis. Alongside its simplicity and non-invasive nature, the FIB-4 index is an effective screening tool for individuals at risk of liver fibrosis. Alternative non-invasive methods for fibrosis assessment, including serum markers and imaging modalities such as Fibrotest, APRI, FibroMeter (Echosens), CirrhoMeter, and acoustic radiation force impulse (ARFI) imaging, have also been validated. These methods enable clinicians to predict significant liver-related events and make informed diagnoses and treatment decisions, further emphasizing the importance of non-invasive approaches in liver fibrosis assessment [33-35].

Comparative analysis of liver scoring systems

Sensitivity and Specificity

The literature presents data on the sensitivity and specificity of diverse liver scoring systems in predicting mortality associated with liver diseases. For instance, in acute-on-chronic liver failure, a study indicated that the CLIF-SOFA score exhibited 78.1% sensitivity and 79.7% specificity. In comparison, the CLIF-C OF score demonstrated 68.8% sensitivity and 91.4% specificity for predicting 28-day mortality [36]. In a meta-analysis assessing test accuracy for scoring systems in decompensated liver cirrhosis, the CTP score, MELD, MELD-Na, and MELD to MESO were identified for mortality prediction. However, specific sensitivity and specificity values were not provided [8]. Moreover, a scoring system aimed at predicting 90-day mortality in in-hospital liver cirrhosis patients yielded a sensitivity of 81.3% and specificity of 50.9% for predicting 28-day mortality through a combination of three variables [9]. Additionally, research on HCC highlighted the HCC-ART score as effective for early diagnosis, boasting a sensitivity of 97% and a specificity of 96% [37]. Furthermore, a study comparing binary predictive scoring systems for posthepatectomy liver failure underscored the importance of reporting sensitivity and specificity to assess the performance of different scoring systems [38]. The sensitivity and specificity of liver scoring systems may fluctuate depending on the specific disease, patient population, and the system utilized, emphasizing the need to consider these factors when interpreting their performance.

Predictive Value for Mortality

Several studies have examined the predictive value of various liver scoring systems for mortality. A meta-analysis focusing on test accuracy in decompensated liver cirrhosis identified the CTP score, MELD, MELD-Na, and MESO as tools commonly used for mortality prediction [8]. However, specific sensitivity and specificity values were not provided in this analysis. Another study conducted on in-hospital liver cirrhosis patients established a scoring system categorizing patients into three risk groups: low risk (score of zero to three) with a 4.1-18.4% probability of death, moderate risk (score of five to six) with a 40.5-54.2% probability of death, and high risk (score of eight to 11) with a 78.1-94.9% probability of death [9]. Additionally, this study demonstrated that a combination of three variables yielded a score with a sensitivity of 81.3% and specificity of 50.9% for predicting 28-day mortality [9].

Another study found that the Chronic Liver Failure-Sequential Organ Failure Assessment (CLIF-SOFA) score exhibited 78.1% sensitivity and 79.7% specificity in acute-on-chronic liver failure. The Chronic Liver Failure Consortium Organ Failure (CLIF-C OF) score demonstrated 68.8% sensitivity and 91.4% specificity for predicting 28-day mortality [36]. Furthermore, research on end-stage liver disease patients revealed that the MELD score outperformed the CTP score in predicting three-month mortality [39]. The predictive value for mortality provided by liver scoring systems can vary depending on the specific disease, patient population, and the scoring system utilized. It is crucial to consider these factors when interpreting the performance of these scoring systems.

Applicability in Different Populations

The utility of liver scoring systems across different populations hinges upon several factors, including the specific disease under consideration, the characteristics of the patient population, and the scoring system utilized. Studies have sought to evaluate the predictive validity of various scoring systems in forecasting mortality in end-stage liver disease [39]. For instance, scoring systems like the CTP score, MELD, MELD-Na, and MESO have been employed to predict mortality in liver cirrhosis patients [39]. However, it is important to note that the sensitivity and specificity of these scoring systems can fluctuate depending on the particular disease and patient demographic. A study focusing on in-hospital liver cirrhosis patients identified three variables to be integrated into a scoring system tailored for individuals with liver cirrhosis, resulting in a score range of zero to 11 [9]. The amalgamation of these three parameters has shown promise as a dependable tool for predicting 90-day mortality in liver cirrhosis patients [9]. Nonetheless, it is crucial to acknowledge factors such as the constrained availability of intensive care unit (ICU) beds and the elevated costs associated with ICU care when employing these scoring systems [9]. Furthermore, another study compared the accuracy of non-invasive scoring systems in gauging the risk of advanced fibrosis in NAFLD [40]. While these scoring systems can aid in mortality prediction and facilitate the identification of requisite care and interventions for liver cirrhosis patients, their application necessitates careful consideration of the specific disease entity and the demographics of the patient population.

Clinical implications and decision-making

Treatment Algorithms Based on Scoring Systems

**Figure 2:**
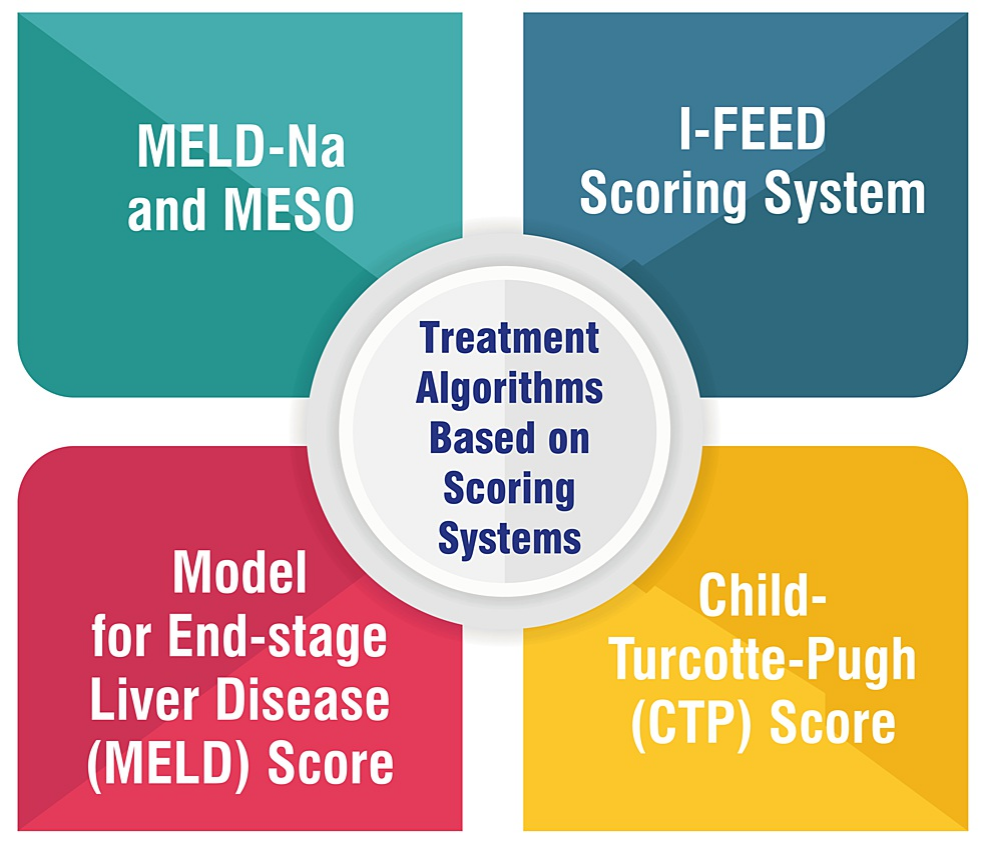
Treatment algorithms based on scoring systems This figure is self-created by the corresponding author.

Several scoring systems play integral roles in managing patients with various liver conditions. First, the I-FEED scoring system is tailored to address patients with impaired postoperative gastrointestinal (GI) function, offering clinicians a structured approach to decision-making in their management [41]. By developing a treatment algorithm rooted in the I-FEED scoring system, clinicians can make informed decisions regarding the optimal management strategies for these patients [41]. Second, the CTP score stands as a widely utilized tool for predicting mortality among individuals with liver cirrhosis [42]. Leveraging the CTP score, clinicians can develop treatment algorithms that facilitate more accurate diagnoses and the selection of effective treatment modalities for patients grappling with liver cirrhosis [42]. Similarly, the MELD score is another prominent scoring system for predicting mortality among liver cirrhosis patients [42]. By employing the MELD score, clinicians can construct treatment algorithms to enhance diagnostic precision and guide treatment decisions for individuals afflicted with liver cirrhosis [42]. Moreover, the MELD-Na and MELD to MESO scoring systems also offer predictive insights into mortality among liver cirrhosis patients [43]. Leveraging these scoring systems, clinicians can develop treatment algorithms geared toward refining diagnostic accuracy and facilitating the selection of effective treatment strategies for individuals grappling with liver cirrhosis [43]. By utilizing these scoring systems and developing treatment algorithms, clinicians can navigate the complexities of liver disease management with greater precision and efficacy. Treatment algorithms based on scoring systems are shown in Figure 2.

Monitoring Disease Progression

Monitoring disease progression in liver cirrhosis necessitates utilizing a variety of non-invasive tests and scoring systems. Current guidelines advocate for the monitoring of NAFLD progression through repeated non-invasive fibrosis tests (NITs) conducted at two- to three-year intervals [44]. These tests serve to assess fibrosis levels and identify patients at heightened risk. Additionally, widely utilized scoring systems such as the Child-Pugh score (CP) and the MELD play pivotal roles in evaluating the severity of liver disease and predicting mortality among cirrhosis patients [9]. In tandem with scoring systems, non-invasive imaging modalities and blood tests are increasingly accessible for liver disease monitoring. These tests aid in gauging the severity of cirrhosis, offering valuable insights into disease progression [45]. However, it is imperative to recognize that while scoring systems and non-invasive tests serve as valuable adjuncts to clinical judgment, they should not supersede it. Clinicians must exercise caution and consider factors such as the restricted availability of ICU beds and the considerable expense associated with ICU care when utilizing these tools [9]. By integrating clinical judgment with the information gleaned from scoring systems and non-invasive tests, clinicians can effectively navigate the intricacies of liver disease management, ensuring optimal patient care.

Patient Counseling and Education

Patient counseling and education play integral roles in the comprehensive management of liver cirrhosis. Given the significant barriers patients with cirrhosis encounter in managing their condition, education is a fundamental clinical competency for effective care [46]. Education initiatives should prioritize equipping patients with a comprehensive understanding of the disease, encompassing its etiology, pathology, and treatment modalities [47]. Additionally, nutritional counseling emerges as a crucial component, aiming to enhance patients' quality of life through dietary modifications tailored to their needs. Continuous monitoring and support are indispensable for patients and their caregivers, facilitating their ability to navigate the challenges associated with managing liver cirrhosis effectively [47]. Clinicians must inform patients about the disease's prognosis and potential complications, fostering informed decision-making and proactive engagement in treatment plans [48]. Encouraging active participation in their treatment regimens empowers patients to take ownership of their health and improves outcomes [47]. It is imperative to underscore that patient education should be individualized to cater to each patient's unique needs and preferences. Clinicians should consider factors such as the patient's health literacy levels and cultural background when delivering education, ensuring that information is communicated effectively and comprehensively [46]. By adopting a patient-centered approach to education and counseling, clinicians can empower patients to manage their condition and actively enhance their overall well-being.

## Conclusions

In conclusion, this review has provided a comprehensive exploration of liver scoring systems for the diagnosis and prognosis of cirrhosis. Through examining essential tools such as the Child-Pugh score, MELD, ALBI score, and FIB-4 Index, we have underscored their crucial role in assessing the severity of liver dysfunction and predicting clinical outcomes. As we recapitulate the key findings, it is evident that these scoring systems offer valuable insights, guiding clinicians in making informed decisions for patient management. We recommend integrating these tools into routine clinical practice, emphasizing early and regular use to facilitate timely interventions and personalized treatment strategies. Moreover, an interdisciplinary collaboration among healthcare professionals is essential for a holistic approach to cirrhosis management, combining expertise from various specialties to ensure a comprehensive evaluation. Future research should focus on refining existing scoring systems, exploring novel biomarkers and imaging techniques, and conducting prospective studies to address the current limitations and enhance the precision of cirrhosis assessment. By embracing these recommendations and actively participating in ongoing research endeavors, the medical community can advance the understanding and management of cirrhosis, ultimately improving patient outcomes and fostering a more practical approach to liver disease.
